# Disinfection and Sterilization Using Plasma Technology: Fundamentals and Future Perspectives for Biological Applications

**DOI:** 10.3390/ijms20205216

**Published:** 2019-10-21

**Authors:** Akikazu Sakudo, Yoshihito Yagyu, Takashi Onodera

**Affiliations:** 1 Faculty of Veterinary Medicine, Okayama University of Science, Imabari, Ehime 794-8555, Japan; 2 Department of Electrical and Electric Engineering, National Institute of Technology Sasebo College, Nagasaki 857-1193, Japan; 3 Research Center for Food Safety, Graduate School of Agricultural and Life Sciences, the University of Tokyo, Bunkyo-ku, Tokyo 113-8657, Japan

**Keywords:** discharge, disinfection, inactivation, sterilization, toxins

## Abstract

Recent studies have shown that plasma can efficiently inactivate microbial pathogens such as bacteria, fungi, and viruses in addition to degrading toxins. Moreover, this technology is effective at inactivating pathogens on the surface of medical and dental devices, as well as agricultural products. The current practical applications of plasma technology range from sterilizing therapeutic medical devices to improving crop yields, as well as the area of food preservation. This review introduces recent advances and future perspectives in plasma technology, especially in applications related to disinfection and sterilization. We also introduce the latest studies, mainly focusing on the potential applications of plasma technology for the inactivation of microorganisms and the degradation of toxins.

## 1. Introduction

Irving Langmuir first coined the term ‘plasma’ in 1927 to describe an ionized gas [1]. Early applications of plasma technology mainly focused in the field of engineering, such as nuclear fusion and plasma etching [2,3,4]. However, over the past 20 years, there has been a plethora of patents and scientific papers describing the microbicidal properties of plasma [5]. Recently accumulated knowledge has led to improvements in the efficiency of the disinfection and sterilization using plasma technology and a growing awareness of its potential utility [5,6,7].

In this review, we summarize the fundamentals of the methods for plasma generation and their application, as well as the efficacy of these disinfection/sterilization methods against various microorganisms. Furthermore, we also discuss the possible future application of this technology in the area of medicine and dentistry as well as agriculture.

## 2. Fundamentals of Plasma and Methods for Its Generation

There are three commonly encountered states of matter: solid, liquid, and gas. When a solid is heated, it transforms into a liquid and then from a liquid into a gas. If enough energy is applied to gas, it becomes an ionized gas known as plasma, which represents the fourth fundamental state of matter [8]. The plasma contains reactive chemical species such as electrons, ions, neutral molecules, and atoms, as well as charged species [9]. In addition, the emission of radiation occurs in the ultraviolet (UV) as well as visible and near-infrared regions during plasma generation.

A state of plasma could be typically classified according to temperature [10,11,12,13,14,15,16,17] (Table 1). In a high-temperature plasma, which is a strong or fully ionized plasma, the temperature of the electrons *T*_e_ and ions *T*_ion_ are the same, so they are in thermal equilibrium with each other by collision due to thermal motion. The gas temperature *T*_gas_ of high-temperature plasma and thermal plasma is too extreme for treating living organisms. Alternatively, in non-thermal plasma, comprising partially ionized plasmas, the temperature of the electrons *T*_e_ is much higher than that of the ions *T*_ion_ and neutrons *T*_n_. The energy transfer of the kinematics of a collision between electrons (light particles) and ions or neutrals (heavy particles) tends to be very slow by elastic collision, but electron-electron collisions readily achieve thermodynamic equilibrium. Therefore, the ionized gas temperature keeps the normally ambient temperature in non-thermal plasma. As a result, the gas temperature of non-thermal plasma remains low, making it suitable for biological applications.

Electrical discharge methods commonly utilized for non-thermal plasma generation in biological applications are generally categorized into one of the following: glow discharge, corona discharge, atmospheric pressure plasma jet (APPJ), dielectric barrier discharge (DBD), micro-hollow cathode discharge (MHCD), DC discharge, pulse discharge, or high/low-frequency discharge (Table 2). The type of discharge depends on the frequency of the power source, such as direct current (DC) and alternating current (AC) discharge, as well as ambient gas pressure, such as low-pressure and atmospheric pressure plasma, and the precise shape and configuration of the electrodes [9]. In addition, the waveform may also affect the type of discharge. Different types of plasma can be used in various biological fields, including disinfection/sterilization.

Non-thermal plasma is easy to obtain under low-pressure conditions because the collisions between electrons, ions, and neutral molecules occur infrequently. Low-pressure plasma can be generated by a low breakdown voltage in a vacuum chamber evacuated with a vacuum pump. Low-pressure plasma systems are important for the manufacture of semiconductor components. Furthermore, research into low-pressure plasma systems has also focused on the decontamination and sterilization of medical devices [23,24]. Although low pressure plasma can generate high concentrations of active species with a uniform glow plasma, it involves high maintenance costs because of the requirement for a vacuum system. Atmospheric pressure plasma requires a high voltage and relatively high temperature due to frequent collisions between electrons and ions accompanying the high particle density [9]. However, it is possible to generate plasma under non-thermal conditions by using a pulse discharge and APPJ [25], DBD [26], and floating electrode barrier discharge (FE-DBD) [27,28], or MHCD [21,29]. These non-thermal conditions allow applications involving exposure of the plasma with tissues such as skin [30].

**Figure 1:**
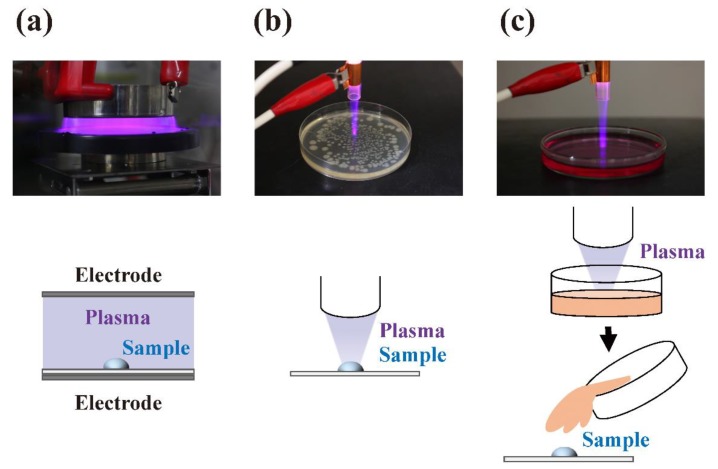
Types of plasma treatment can be classified as follows. (**a**) Discharging area is in contact with the sample, or (**b**) the sample is in contact with plasma transferred to the target site from the discharging area. Alternatively, (**c**) solutions previously subjected to plasma treatment could be used as reagents to apply to samples. Photographic images of the different modes of treatment are shown in the upper section with corresponding illustrations in the lower section.

Similarly, non-thermal plasma can be used to disinfect agricultural products and medical devices with relatively little impact on their structural integrity [31,32]. Alternatively, the plasma could be transferred to a target site where the object for treatment is located using a plasma afterglow (Figure 1). Indirect treatment using solutions treated with plasma, known as “plasma-activated water (PAW)” [33], “plasma-activated medium (PAM)” [34], “plasma-stimulated medium (PSM)” [35], “plasma-treated water (PTW)” [36,37], “plasma-treated phosphate-buffered saline (pPBS)” [38], or “non-thermal plasma-conditioned media (NTP media)” [39], is also possible. The constituents in these solutions react with samples and act as disinfectants [40] or anti-cancer agents [41]. In cases where samples are in contact with plasma bulk in the discharging area, plasma components such as UV radiation and reactive chemical species directly interact with the samples. Thus, short-life reactive chemical species, such as reactive oxygen species (ROS) and reactive nitrogen species (RNS), efficiently interact with the sample components [42]. By contrast, in cases where the sample is in contact with plasma bulk away from the discharging area, the contribution of UV radiation is significantly lower. In addition, there is a greatly reduced concentration of reactive chemical species in the post discharging area due to their short half-life at ambient temperature. In the case of plasma-treated solutions, freezing can extend the storage time and minimize the loss of reactive chemical species [37].

## 3. Inactivation of Microorganisms by Plasma

Some microorganisms such as bacteria, viruses, and fungi act as pathogens and cause diseases. There is a resistance hierarchy of microorganisms against disinfection/sterilization that can be divided into the following five categories: most resistant, highly resistant, intermediately resistant, less resistant, and very susceptible [43]. The most resistant infectious agents are prions (proteinaceous infectious particles), which are the causative agents of prion diseases, such as the Creutzfeldt–Jakob disease (CJD) in humans, bovine spongiform encephalopathy (BSE) in cattle, and chronic wasting disease (CWD) in cervids (deer family). Bacterial spores, protozoan oocysts, and helminth eggs are categorized as highly resistant microorganisms. Intermediately resistant microorganisms include mycobacteria, protozoan cysts, small non-enveloped viruses, and fungal spores. Vegetative bacteria, protozoa, helminths, fungi, and algae, as well as large non-enveloped viruses are less resistant. Enveloped viruses such as human immunodeficiency virus (HIV) are generally highly susceptible to various disinfectants.

However, in reality, the situation is more complex. For example, although the enveloped virus HIV is very sensitive to disinfection and is rapidly inactivated even at room temperature without any treatment, the influenza virus (another enveloped virus) can remain infectious for up to 48 h. Orthopoxviruses (e.g., smallpox, vaccinia) and filoviruses (e.g., Ebola virus, Marburg virus) are all enveloped viruses that remain infectious for up to several weeks. Furthermore, susceptibility against biocides depends on the environment where the microorganisms are present (e.g., blood, serum, spinal fluid, saliva). Certain soils can prevent drying and stabilize the viral structure, extending the survival time of viruses. For example, viruses normally interact with external materialss including proteins, lipids, salts, and cell debris. In other cases, viruses cause the aggregation of host cells [44] or make aggregates themselves [45], which could reduce the virucidal effect of disinfectants. Therefore, the susceptibility of microorganisms to disinfection/sterilization should be examined under a range of different conditions.

The most impressive results in terms of microbicidal activity have come from studies using bacteria, including bacterial spores [4,5,6,7,8,9,10,11,12,13,14,15,16,17,18,19,20,21,22,23,24,25,26,27,28,29,30,31,32,33,34,35,36,37,38,39,40,41,42,43,44,45,46,47,48,49]. Although some bacteria can form biofilms in certain environments, this does not prevent their successful inactivation by plasma treatment [50,51]. Furthermore, the recent emergence of drug-resistant pathogens is now a serious public health concern that has been acknowledged by both the World Health Organization (WHO) [52] and the US Centers for Disease Control (CDC) [53]. The extensive and indiscriminate use of antibiotics may have altered the environmental microbiome, contributing to the emergence of drug-resistant bacteria [54]. Consequently, there is an urgent requirement to devise novel methods to eliminate these multidrug-resistant bacteria from the food production process. Plasma treatment is an especially promising method because the mechanism of bactericidal action is unlikely to differ between multidrug-resistant and normal bacteria. The main mechanisms of bactericidal action in plasma are thought to involve exposure to reactive chemical species for which multidrug-resistant bacteria are unlikely to be resistant [55]. Furthermore, plasma pre-treatment enhances the sensitivity of methicillin-resistant *Staphylococcus aureus* to antibiotics [56].

**Figure 2:**
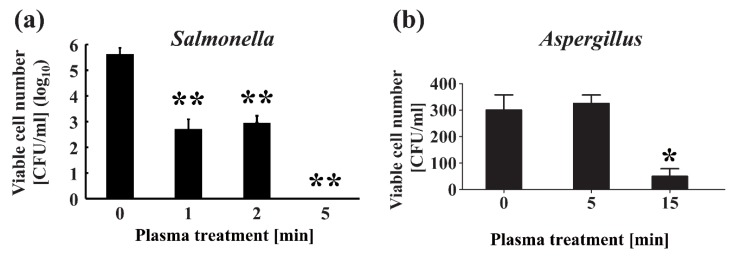
Direct plasma treatment inactivates bacteria and fungi. (**a**) A sample of *Salmonella* was treated with plasma using the BLP-TES (bi-polar and low-pressure plasma-triple effects sterilization) device, which generates nitrogen gas plasma using a fast high-voltage pulse by a static induction (SI) thyristor power supply, for the indicated time. Colony-forming units (CFU) per ml of culture reduced with the plasma treatment in a time-dependent manner. (**b**) Viable cell numbers of *Aspergillus* reduced after plasma treatment using the BLP-TES device. Differences where * *p* < 0.05 and ** *p* < 0.01 versus control (0 min) were considered significant. Modified from Maeda et al. 2015 [55] and Sakudo et al. 2017 [57] with permission from Elsevier.

Several reports suggest that plasma can be effective in inactivating fungi [57,58,59]. However, our own investigations have shown that the viable cell number of *Aspergillus brasiliensis* was not significantly impacted after 5 min plasma treatment using a nitrogen gas plasma device, BLP-TES (bi-polar and low-pressure plasma-triple effects sterilization) [57], whereas *Salmonella enterica* serovar Abony was completely inactivated after employing the same treatment regime [55] (Figure 2). Indeed, a 15 min treatment with nitrogen gas plasma was required to reduce the viability of *A. brasiliensis*. Thus, by comparison to bacteria, extended treatment time with nitrogen gas plasma must be used to inactivate fungi.

The resistance of fungi to plasma treatment has also been studied. Soušková et al. reported the susceptibility of fungi is different between species, including *Aspergillus oryzae*, *Cladosporium sphaerospermum,* and *Penicillium crustosum* despite no significant differences in susceptibility against plasma generated by corona discharge among bacteria, including *Escherichia coli* and *Staphylococcus epidermidis* [58,59,60]. Among these fungi, *Aspergillus* displayed the greatest resistance to plasma inactivation, possibly due to the presence of spores. Therefore, the resistance of fungi to plasma treatment appears to be related to spore generation.

**Figure 3:**
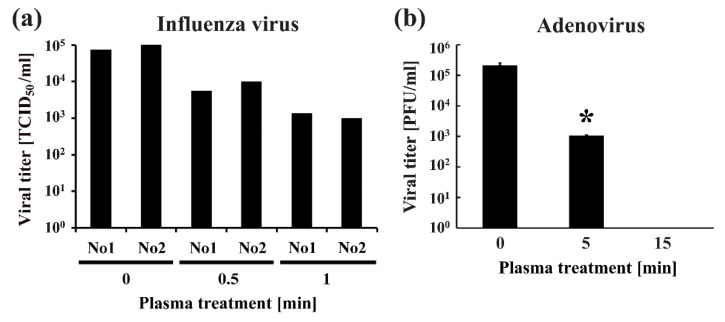
Direct plasma treatment inactivates viruses. (**a**) Viral titers of median tissue culture infectious dose (TCID_50_) per ml were calculated in duplicate (No 1 and No 2) for the influenza virus. The TCID_50_ values reduced after treatment with low-pressure nitrogen gas plasma using the BLP-TES device. (**b**) Nitrogen gas plasma treatment with the BLP-TES device resulted in a decrease in viral titer [plaque forming units (PFU) per ml] of adenovirus. Differences where * *p* < 0.05 versus control (0 min) were considered significant. Cited from Sakudo et al. 2013 [61] and Sakudo, Toyokawa, and Imanishi 2016 [62] under the terms of the Creative Commons Attribution license.

Several studies have investigated the effect of plasma on the inactivation of both enveloped and non-enveloped viruses. Representative studies showed that nitrogen plasma generated by BLP-TES inactivated enveloped viruses, such as the influenza virus [61] and respiratory syncytial virus (RSV) [42], as well as non-enveloped viruses, such as the adenovirus [62] (Figure 3). In addition, there are several studies using bacteriophages as model objects of viral inactivation by plasma [63,64,65].

Furthermore, a DBD plasma torch inactivated the non-enveloped virus, feline calicivirus [66]. Inactivation of viruses was achieved by a relatively short exposure to plasma. According to the U.S. Environmental Protection Agency (USEPA) “Guide Standard and Protocol for Testing Microbiological Water Purifiers,” the minimum performance standards of the inactivation efficiency are a six-log reduction/inactivation of bacteria, or a four-log reduction/inactivation of viruses [67]. Treatment using nitrogen gas plasma generated by BLP-TES showed an approximate two-log reduction in influenza virus titer after 1 min and four-log reduction of virus titer of adenovirus within 4 min [61,62]. A 1-min treatment with the DBD plasma torch resulted in a greater than two-log reduction of virus titer for feline calicivirus [66]. Lengthening the treatment to 2 min reduced the viral titer to an undetectable level (3.81 × 10^4^ ± 1.58 × 10^3^ median tissue culture infectious dose (TCID_50_)/_mL_ at 0 min; below the detection limit at 2 min). These results suggest that the 2 min treatment meets the performance standards set by USEPA as outlined earlier. However, as far as the authors are aware, there are a limited number of studies on the inactivation of plant viruses using plasma [68]. Furthermore, there are no published studies on the plasma inactivation of viroid’s, which are infectious RNAs that cause plant diseases. Indeed, to develop a plasma disinfection system for the agricultural sector, it would be necessary to determine the effectiveness of this technology on a range of plant pathogens.

**Figure 4:**
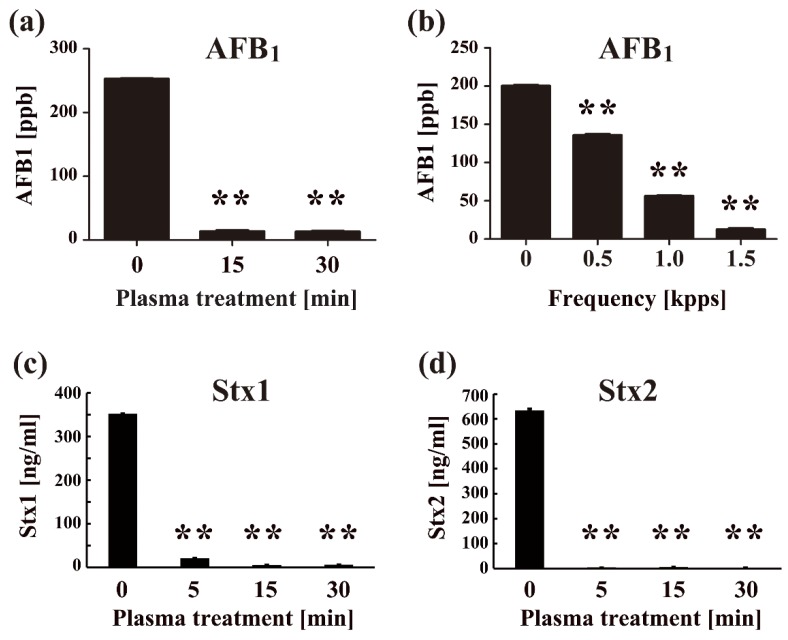
Direct plasma treatment inactivates toxins. Quantitative measurement of aflatoxin B_1_ (AFB1) (**a**,**b**), Shiga toxin 1 (Stx1) (**c**), Shiga toxin 2 (Stx2) (D) after low-pressure nitrogen gas plasma treatment with BLP-TES at 1.5 kpps for the indicated times (**a**,**c**,**d**) and at 0–1.5 kpps for 15 min (**b**) was performed by an enzyme-linked immunosorbent assay (ELISA) using an MytoJudge Total Aflatoxin kit (NH Foods Ltd.) and a RIDASCREEN^®^ Verotoxin kit (R-Biopharm AG, Darmstadt). Differences where ** *p* < 0.01 versus control (0 min) were considered significant. (**a**) and (**b**) are cited from Sakudo et al. [57] with permission from Elsevier, while (**c**) and (**d**) are cited from Sakudo et al. [74] under the terms of the Creative Commons Attribution 4.0 International license.

Plasma is also effective against other microorganisms besides the ones mentioned above. For example, plasma inactivation of the yeast-like algae *Prototheca zopfii* [69] and water-borne helminth *Schistosoma japonicum* [70], *Acanthamoeba* species (spp.), and other ocular pathogens, as well as water-borne protozoan enteroparasite *Cryptosporidium parvum* when combined with pulsed UV [71] was confirmed. These results suggest that plasma has the potential to inactivate cysts and protozoal oocysts as well as trophozoites of protozoon parasites. Although prions are known to be the most resistant pathogens, they are nonetheless inactivated by radio-frequency (RF) plasma treatment using an Ar/O_2_ gas mixture [72] and by plasma from a microwave discharge [73]. In addition, plasma treatment can efficiently degrade toxins produced by both bacteria and fungi (Figure 4).

Thus, plasma has applications not only for disinfection/sterilization but also for the degradation of toxins [75]. This includes not only exotoxins but also endotoxins, lipopolysaccharides (LPS) by inactivating their lipid A [76]. Consequently, plasma technology is a novel advanced disinfection/sterilization system that can simultaneously inactivate pathogens and their associated toxins.

The inactivation mechanisms of action of plasma remain to be determined [5,6,31,77]. The mechanisms depend on the types of gases used to generate the plasma. In the case of nitrogen gas plasma, at least three major mechanisms (reactive chemical species, UV radiation exposure, and electric fields) are thought to be involved [31]. In addition, etching effects may also contribute, especially in the case of oxygen plasmas [78]. Specifically, shrinking of bacterial spores were observed in oxygen gas plasma-treated spores but not in nitrogen gas plasma-treated spores [79,80,81].

Overall, reactive chemical species, UV radiation, and electric fields contribute to the antimicrobial effects of plasma, depending on the type of gases as well as the methods employed to generate the plasma. Reactive chemical species seem to be the principal inactivation factor in most cases, although this may vary depending on the method of plasma generation and whether the sample is exposed to direct or indirect plasma treatment.

In addition, inactivation mechanisms may vary depending on the target microorganism. However, it should be noted that most studies on the inactivation mechanisms of plasma have been conducted using bacterial spores. Therefore, further studies are required on the inactivation of various microorganisms using plasma to understand the underlying mechanisms involved fully.

## 4. Future Perspectives in Agriculture and the Food Industry

There are extensive applications of plasma technology in the field of agriculture and in the food sector. For example, plasma technology could be applied to the disinfection of foods, packaging materials and equipment as well as agricultural sources such as seeds, fertilizer, water, and soil.

Agricultural products, such as fruits and vegetables, are prone to contamination from agricultural sources, including seeds, fertilizers, water, and soil. Moreover, the agricultural products come into contact with dust, insects, animal feces, field workers, and equipment during pre-harvest/harvest, transport, packaging and food processing stages of the supply chain. These individual risk factors may contribute to a microbial hazard. The application of innovative disinfection techniques, including plasma technology, may help reduce the potential risk from these factors. A recent review has shown that food products subjected to plasma disinfection are becoming widespread, which include fresh fruits, vegetables, dry fruits, nuts, seeds, spices, eggshells, as well as protein products such as meat and cold cuts [82]. However, further advances in plasma disinfection technology are required before this method can be applied to the food industry (e.g., food processing and distribution system as well as agricultural products).

**Figure 5:**
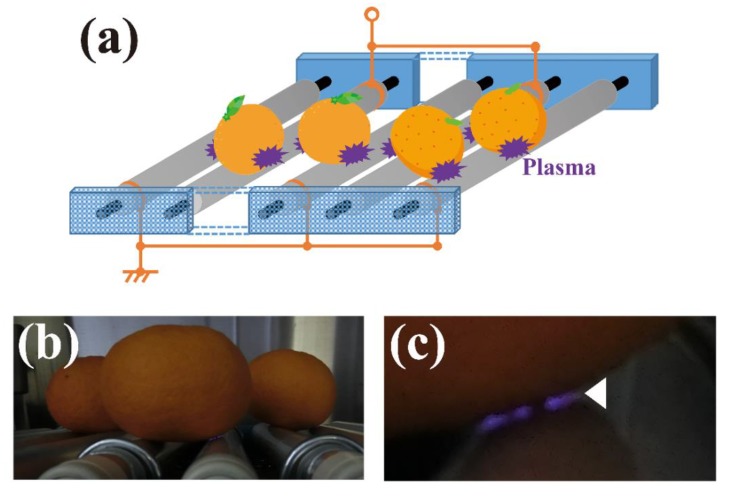
(**a**) Schematic representation of a roller conveyer plasma device for the continuous disinfection of fruits and vegetables using atmospheric pressure plasma. As an example, oranges are shown. (**b**) Oranges on rollers during operation of the device. (**c**) Enlarged image of (**b**) showing the plasma (Arrow) generated between the orange and roller during operation of the device. The image is modified from Toyokawa et al. 2017 [83] with permission from Elsevier.

We anticipate the wide-ranging application of plasma technology in the field of agriculture once the equipment has been fully developed. The current priority is the development of an efficient open-air system suitable for disinfecting both large objects and high numbers of samples. Moreover, such a device will be readily scalable. Based on this background, we recently developed a novel roller conveyer plasma device (Figure 5).

This plasma device is well suited to the disinfection of fruits and vegetables during sorting on rollers [83]. The apparatus is an original design that generates atmospheric plasma by the mechanism of DBD. This unique plasma apparatus is composed of rolling electrodes comprising a plastic rod (diameter = 30 mm) covered with a thin aluminum and silicon sheet positioned at an interval of 50 mm between the high -voltage electrode and earth electrode. The high-voltage electrode is then connected to an alternating power supply. Plasma is generated in the silicon sheet when electrically conductive samples, such as fruits and vegetables as well as metals, make contact with both the high-voltage electrode and earth electrode. Our findings suggest the device could have practical applications for the disinfection of agricultural products during the sorting process on rollers. Disinfection of *Xanthomonas campestris* p.v. *campestris*-contaminated cabbage leaves using the roller conveyer plasma device has been achieved. In addition, our preliminary study has shown that the surface of *Penicillium*-contaminated oranges could be disinfected using the device (Sakudo and Yagyu, unpublished results). Nonetheless, to achieve broad applicability with a variety of agricultural food products, the device needs to be scaled-up and its performance fully evaluated.

In addition, a critical factor to consider is the safe application of this novel technology. The European Commission stated that there are no restrictions in the regulations regarding the use of plasma as an electronic preservative practice for organic foods [84]. However, plasma treatment of aqueous solutions can potentially generate hydrogen peroxide, nitrates, and nitrites [85,86]. These compounds might react to form other toxic compounds such as peroxynitrous acid. Therefore, a comprehensive evaluation of the effect of plasma on foods and human health is necessary before this new disinfection technology can be fully utilized. Indeed, a range of applications of plasma technology in the field of agriculture is currently being assessed.

Recently, the use of plasma technology has been reported to enhance seed germination and the growth of plants [87]. In addition, the removal of volatile organic compounds, such as ethylene gas, by plasma treatment may be useful during the transportation of agricultural products in containers [88]. Therefore, the application of plasma technology could also contribute to higher crop yields as well as the preservation of foods.

## 5. Future Perspectives in Medicine and Dentistry

Sterilization of medical instruments contaminated with pathogens is crucial in preventing secondary infections. Currently, medical instruments are sterilized by autoclaving, gamma-ray treatment, UV exposure, and the use of gases such as ethylene oxide, hydrogen peroxide, formaldehyde, peracetic acid [43]. Each sterilization method has both advantages and disadvantages. Autoclaving is relatively quick, highly penetrative, and generates no toxic residues, but temperatures of 121 °C could damage the material being sterilized. Treatment with gamma-rays is highly penetrative, and involves low temperatures with no associated residues, but it could induce changes in the properties of the materials and is a relatively slow process. UV treatment is fast, low cost with no toxic residues, and involves low temperatures, but the effectiveness of the sterilization is poor and may result in damage to the material. Chemical treatments are low temperature and effective, but these procedures generally involve the use of toxic gases that may be carcinogenic and flammable. Furthermore, these gases sometimes induce unwanted biochemical changes. Although novel techniques have been developed, such as chemical treatment with supercritical carbon dioxide as well as freeze-drying and other methods, these procedures are often ineffective and may damage the material being sterilized. Thus, it is necessary to evaluate each sterilization method for a particular purpose carefully. Plasma technology is a promising new method that enables rapid processing at low temperatures without any associated chemical residues [6]. Plasma is effective against a broad spectrum of pathogens, including bacterial spores and prions, both of which display a high level of resistance to chemical and physical treatments [47,89].

The potential of plasma technology in medical and dental applications is extremely broad. As well as disinfection/sterilization of medical and dental devices, the technology could be used to treat beds, desks, and floors [90]. Plasma technology may also have therapeutic potential [91,92,93]. Therapeutic uses include the treatment of skin diseases [94], blood coagulation [95] as well as dental treatment [96] and applications in dermatology such as chronic wound healing [97]. Recently, the potential application of plasma technology as a novel anticancer therapy has been assessed [98]. Induction of cancer cell death by both direct and indirect exposure of plasma has been reported [99,100]. However, as actual therapy, plasma is often difficult to apply to cancer cells. As an alternative approach, a plasma-irradiated medium (PAW, PAM, PSM, PTW, pPBS, NTP media) has been used for the treatment of cancer cells and tumor tissues that resulted in cell killing and tumor-shrinking effects [41,101,102,103]. In addition, PAM is reported to inhibit the MAP (Mitogen-activated protein) kinase (MAPK) pathway, which is an important signaling pathway for cell proliferation [104]. Cell death is induced by the suppression of these signaling cascades [105]. Moreover, ROS and RNS in plasma are key factors for the induction of cancer cell death, although the mechanisms of action have not been fully elucidated [106,107].

## 6. Conclusions

**Figure 6:**
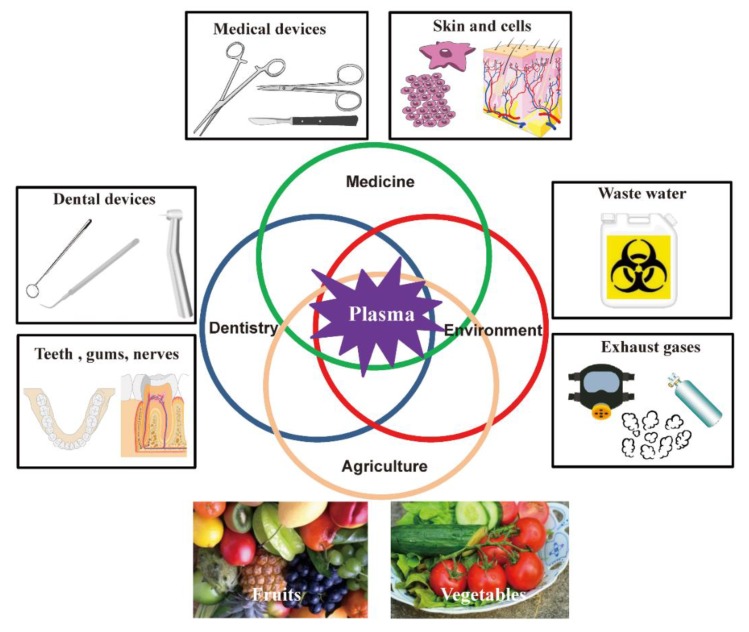
Recent and potential applications of plasma disinfection technology in the field of agriculture, medicine, dentistry, and environment. In the agricultural field, plasma technology could be applied to the disinfection of foods and packaging materials as well as agricultural sources such as seeds, fertilizers, waters and soils. In the medical field, plasma is useful for disinfection/sterilization of medical devices, as well as the degradation of toxins and other pathological contaminants. Potential applications of this technology also includes skin antisepsis as well as the treatment of pathogen-based skin diseases. In dentistry, plasma treatment has been used for microbicidal decontamination, including root canal disinfection and tooth disinfection. Plasma technology may also be utilized in the environmental field, including cleaning of wastewater as well as the treatment of exhaust gases.

In conclusion, plasma disinfection covers almost all of the resistance hierarchy of microorganisms. The susceptibility to the plasma of microorganisms categorized as being most resistant, highly resistant, intermediate resistant, less resistant, and very susceptible, have already been studied. Therefore, the applicability of plasma technology in disinfection/sterilization is potentially wide-ranging (Figure 6). However, to date, no studies investigating plasma treatment of viroids have been reported. Investigation of the effect of plasma on various microorganisms would potentially contribute to further expanding the applicability of this technology. In addition to the field of agriculture and medicine, plasma technology has also been utilized in a range of environmental applications, including water purification and remediation, as well as the treatment of exhaust gases [108,109]. We anticipate that the utilization of this technology will continue to expand.

Finally, it should be mentioned that an increase in the disinfection efficiency and improved cost performance is required before the true potential of plasma technology can be fully realized. This may be achieved by optimization of the plasma generating conditions, including the use of different gas mixtures and careful control of the relative humidity as well as plasma generation methods.

## Figures and Tables

**Table 1:** Typical classification of plasma [10,17].

Classification	Temperature [K]	Electron Density [m^−3^]	Discharge Type	Examples
High-temperature plasma (Equilibrium plasma)	*T*_e_ ≈ *T*_ion_ ≈ *T*_gas_ = 10^6^–10^8^	*n*_e_ ≥ 10^20^	Laser fusion Tokamak	Fusion plasma for energy
Thermal plasma (Quasi-equilibrium plasma)	*T*_e_ ≈ *T*_ion_ ≈ *T*_n_ ≈ *T*_gas_ ≤ 2 × 10^4^	*n*_e_ ≥ 10^20^	Arc plasma, Plasma torch, Radio-frequency (RF) Plasma, Microwave plasma etc.	Radiation, welding and cutting, Waste treatment, Material processing, etc.
Non-thermal plasma (Non-equilibrium plasma)	*T*_e_ ≥ *T*_ion_ ≥ *T*_n_ ≈ *T*_gas_ = 300–1000	*n*_e_ ≈ 10^10^	Glow discharge, Corona discharge, atmospheric pressure plasma jet (APPJ), dielectric barrier discharge (DBD), micro-hollow cathode discharge (MHCD), Plasma needle, Low-pressure plasma etc.	Ozonizer, Plasma medicine, Volatile organic compound (VOC) treatment, Plasma agriculture, Surface modifications (coating, etching, activation, cleaning, nitration, etc.), Illumination (plasma screen, fluorescent lamps, etc.)

*T*_e_ = electron temperature, *T*_ion_ = ion temperature, *T*_gas_ = gas temperature, *n*_e_ = electron density.

**Table 2:** Various types of electrical discharge methods for non-thermal plasma generation.

Discharge Type *	Representative Conditions (V, A, Freq, Gas)	Pressure	Gas Temperature	Application	References
Direct current (DC) corona discharge	5–30 kV direct current (DC) (positive and negative); 10–250 µA; dry or wet; O_2_, N_2_, Ar, He at 10 L/min	1 atm	Room temperature	Biomedical applications	[18]
Atmospheric pressure plasma jet (APPJ) microwave	P = 2.5 W; 2.45 GHz; He/O_2_/N_2_ at 2.0/1.2/1.5 L/min	1 atm	Max. 50.8 °C on a dentin surface; 20 °C on an agar surface	Biomedical applications	[19]
Dielectric barrier discharge (DBD) (Flexible sheet-type)	±2.5 kV; 5 kHz; air, humidity 64.4%	1 atm	Approximately 50 °C	Biomedical applications	[20]
Micro-hollow cathode discharge (MHCD) jet	1.5–2.5 kV DC; 20 mA; air (0.1–8 L/min)	>1 atm	Room temperature (220 mL/min); >55 °C (5 mm from nozzle, 220 mL/min)	Medical applications	[21]
Pin-to-hole spark discharge (PHD) plasma	4 kV DC; average ~1.8 J/pulse	1 atm	9030 ± 320 K (by Boltzmann calculation)	Medical applications (wound healing)	[22]

* These types of electrical discharge are for atmospheric pressure plasmas.

## Abbreviations

AC	Alternating current
APPJ	Atmospheric plasma jet
BLP-TES	Bi-polar and low- pressure plasma-triple effects sterilization
BSE	Bovine spongiform encephalopathy
CDC	Centers for Disease Control
CJD	Creutzfeldt-Jakob disease
CWD	Chronic wasting disease
DBD	Dielectric barrier discharge
DC	Direct current
ELISA	Enzyme-linked immunosorbent assay
FE-DBD	Floating electrode barrier discharge
HIV	Human immunodeficiency virus
LPS	Lipopolysaccharides
MAP	Mitogen-activated protein
MAPK	MAP kinase
MHCD	Micro hollow cathode discharge
*n*_e_	Electron density
NTP	Non-thermal plasma
PAM	Plasma-activated medium
PAW	Plasma-activated water
PHD	Pin-to-hole spark discharge
pPBS	Plasma-treated phosphate-buffered saline
PSM	Plasma-stimulated medium
PTW	Plasma-treated water
RF	Radio-frequency
RNS	Reactive nitrogen species
ROS	Reactive oxygen species
RSV	Respiratory syncytial virus
*T*_e_	Electron temperature
*T*_gas_	Gas temperature
*T*_ion_	Ion temperature
*T*_n_	Neutron temperature
USEPA	U.S. Environmental Protection Agency
UV	Ultraviolet
VOC	Volatile organic compound
WHO	World Health Organization
